# Increased prevalence of depression in South Korea from 2002 to 2013

**DOI:** 10.1038/s41598-020-74119-4

**Published:** 2020-10-12

**Authors:** Ga Eun Kim, Min-Woo Jo, Yong-Wook Shin

**Affiliations:** 1 Department of Psychiatry, College of Medicine, Ewha Womans University, Seoul, Korea; 2 Department of Preventive Medicine, University of Ulsan College of Medicine, 88 Olympicro-43gil, Songpa-gu, Seoul, 05505 Korea; 3 Department of Psychiatry, Asan Medical Center, University of Ulsan College of Medicine, 88 Olympicro-43gil, Songpa-gu, Seoul, 05505 Korea

**Keywords:** Epidemiology, Epidemiology, Epidemiology, Epidemiology

## Abstract

South Korea has one of the highest suicide rates among countries. However, the prevalence of depression in South Korea has been reported to be much lower than in other countries. The current study aims to estimate the prevalence of major depressive disorder using a large representative sample of the South Korean population. The prevalence of depression in a sampled population of one million individuals increased from 2.8% in 2002 to 5.3% in 2013; it was found to increase with the age of the population, and was higher in females than in males for most age groups. A Cox’s proportional hazard model showed that suicide risk was significantly higher in people with depression (hazard ratio [HR] 3.79, 95% CI 3.14–4.58) than those without depression. It was also significantly higher in older people (HR 1.52, 95% CI 1.36–1.70) than in younger people, and in males (HR 2.45, 95% CI 2.02–2.96) than in females. Furthermore, higher income groups were at lower suicide risk as compared to lower income groups (HR 0.88, 95% CI 0.80–0.95). This study using the large representative sample data provided evidence that increased prevalence of depression contributed to the increased risk of suicide in South Korea during the recent decade.

## Introduction

In 2017, depression was the third leading cause of years lived with disability after low back pain and headache disorders^1^. Depression is also a major risk factor for suicidal mortality in the general population^2,3^. South Korea has the highest suicide rate among Organization for Economic Co-operation and Development (OECD) countries, about 2.4 times higher than the average suicide rate of other OECD countries^4,5^.

However, despite the high suicide rate, the prevalence of depression in South Korea has been reported to be much lower than in other countries. Cho et al. reported that the annual prevalence of major depressive disorder was 1.7%, 2.5%, and 3.1% in 2001, 2006, and 2011, respectively^6–8^. These values are lower than those of high-income countries (5.5%) and low- to middle-income countries (5.9%) as observed in a cross-national study^9^, and lower than average values of various countries (7.2%) reported in a recent meta-analysis^10^. In the previous cross-sectional national surveys reporting the prevalence of depression in South Korea, the study populations were convenient and different in each study, and the number of subjects in the target population was small, which makes their results less representative of the general population of South Korea.

The access rate to health care services for depression has been reported to be low. One study reported that the percent annual treatment rate for major depressive episodes was 39.2%^11^. The mental health treatment gap for major depression was 56.3%^12^, with 38% of the patients receiving antidepressant pharmacotherapy at primary care facilities^13^. The usage of antidepressants is increasing globally and the association between antidepressants and suicidal risk is still a matter of debate^14,15^. However, there has been little research on the prevalence of antidepressant treatment with respect to depression in South Korea.

The National Health Insurance Service-National Sample Cohort (NHIS-NSC) is a representative sample database having the clinical record of about one million patients randomly selected from approximately 97% of the overall population of South Korea from 2002 to 2013^16^. Increasing number of researchers have reported the prevalence of various diseases using this database^17–19^ since this large-sized data provides a unique opportunity to examine the epidemiological characteristics of the whole population of South Korea, free from recall bias or nonresponse that are frequently found in survey studies^20,21^. This study aimed to estimate the annual prevalence and incidence of major depressive disorder in the South Korean population over the last decade. We also investigated the rate of prescription of antidepressants. To understand the influence of depression on suicidal mortality^22^, we examined suicidal risk in populations both with and without depression.

## Results

Table 1 shows the epidemiology of total population and patients with depression in 2013 (refer to Lee et al.^16^ for more detailed socio-demographic characteristics of the population from the NHIS-NSC database). The table shows that depression was more prevalent in females than in males, and in older people than in young people. The prevalence of depression was significantly higher in people from the lowest or highest income percentiles than in those belonging to the middle-ranged income percentile. It was also significantly higher in people from non-metropolitan areas than in those from metropolitan areas. Although both these comparisons showed statistical significance, the effect size was small (Table 1).

**Table 1:** Epidemiology of total population and patients with depression in 2013.

	Total	Depression	p	Effect size (Cramer’s V)
**Sex**			< 0.001	0.06
Male	507,289	19,700 (3.88%)		
Female	507,441	34,550 (6.81%)		
**Age**				0.19
0–9	212,768	1463 (0.69%)		
20–39	287,875	7700 (2.67%)		
40–59	336,151	19,258 (5.73%)		
60–79	152,412	21,144 (13.87%)		
> 80	25,524	4685 (18.36%)		
Household income*, percentile, n (%)			< 0.001	0.03
< 20	169,588	11,707 (6.90%)		
21–50	236,253	11,412 (4.83%)		
51–80	324,479	15,208 (4.69%)		
> 81	284,410	15,923 (5.60%)		
Residential area**, n (%)			< 0.001	0.01
Metropolitan	465,853	23,284 (5.00%)		
Nonmetropolitan	548,877	30,966 (5.64%)		

*Income group was divided into 4 groups according to the information of insurance premium group: Group 1 included people from Medicaid and 20%tile of insurance premium; Group 2 included people of 21–50%tile of insurance premium; Group 3 included people of 51–80%tile of insurance premium; Group 4 included people of 81–100%tile of insurance premium.

**The region was divided into two groups, metropolitan and countryside. Metropolitan included the capital city of South Korea (Seoul) and six metropolitan cities such as Busan, Incheon, Daegu, Daejeon, Gwangju, and Ulsan. Other regions were allocated in the nonmetropolitan.

**Figure 1:**
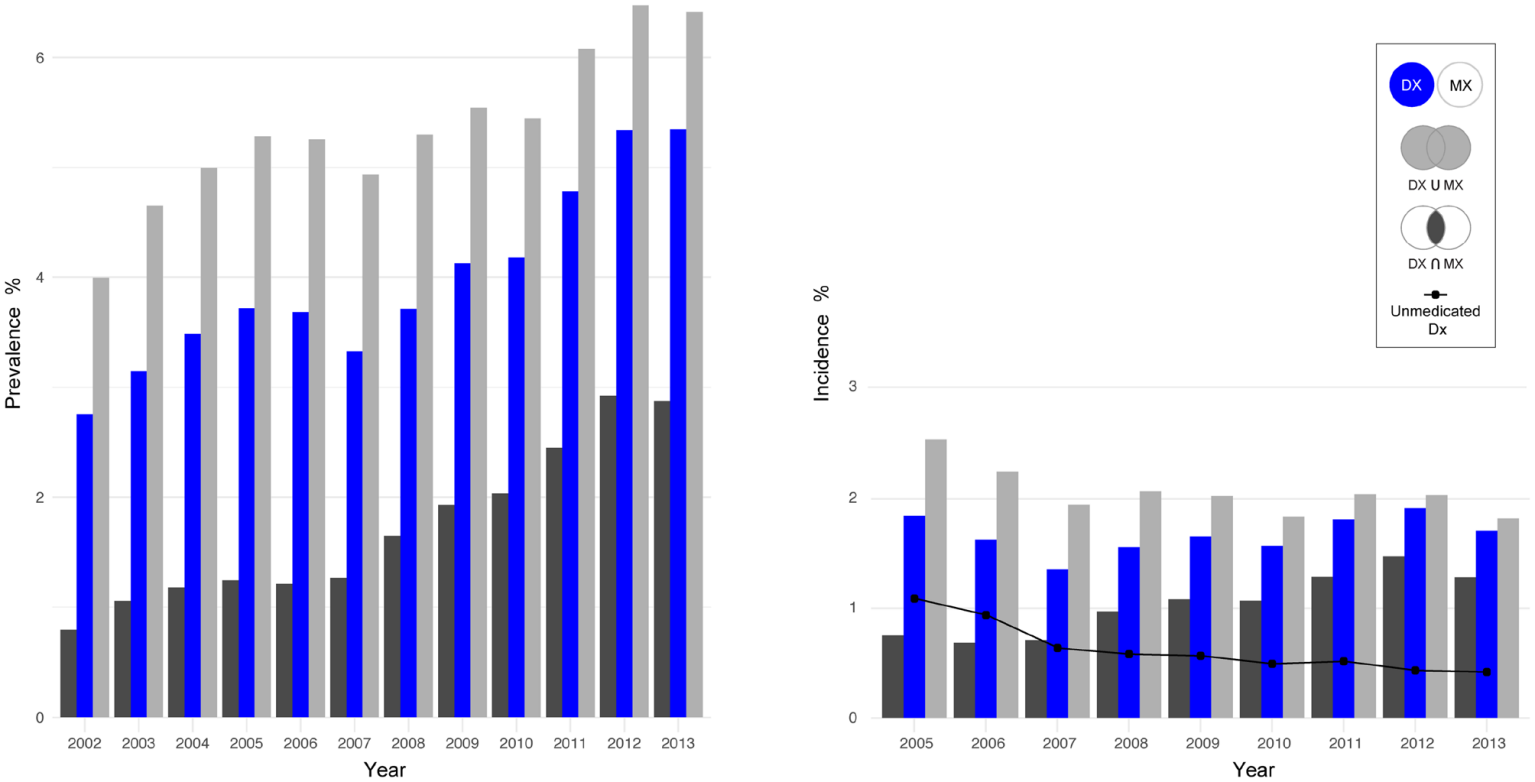
Age- and sex-standardized prevalence and incidence of depressive disorder in South Korea.

Figure 1 shows annual prevalence and incidence of depression during the study period, from 2002 to 2013. The prevalence of depression in the total population in 2002 and 2013 was 2.8% and 5.3%, respectively (‘Dx’ group). The change in the prevalence of depression diagnosis and history of antidepressant treatment (‘Dx and Mx’ group), and that of depression diagnosis or history of antidepressant treatment (‘Dx or Mx’ group), were similar to that of Dx group; the prevalence increased during the study period, with the exception of a minor temporary decrease in 2007 and 2013 in the ‘Dx’ and ‘Dx or Mx’ groups. Among those diagnosed with depression, the proportion of people who were prescribed antidepressants increased steadily during the study period. While about a quarter of the people diagnosed with depression were prescribed antidepressants in 2002, close to half were prescribed antidepressants in 2013. There was a stationary pattern of change in the incidence of depression, although there were slight fluctuations during the study period. Notably, the number of ‘Unmedicated Dx’ individuals decreased.

**Figure 2:**
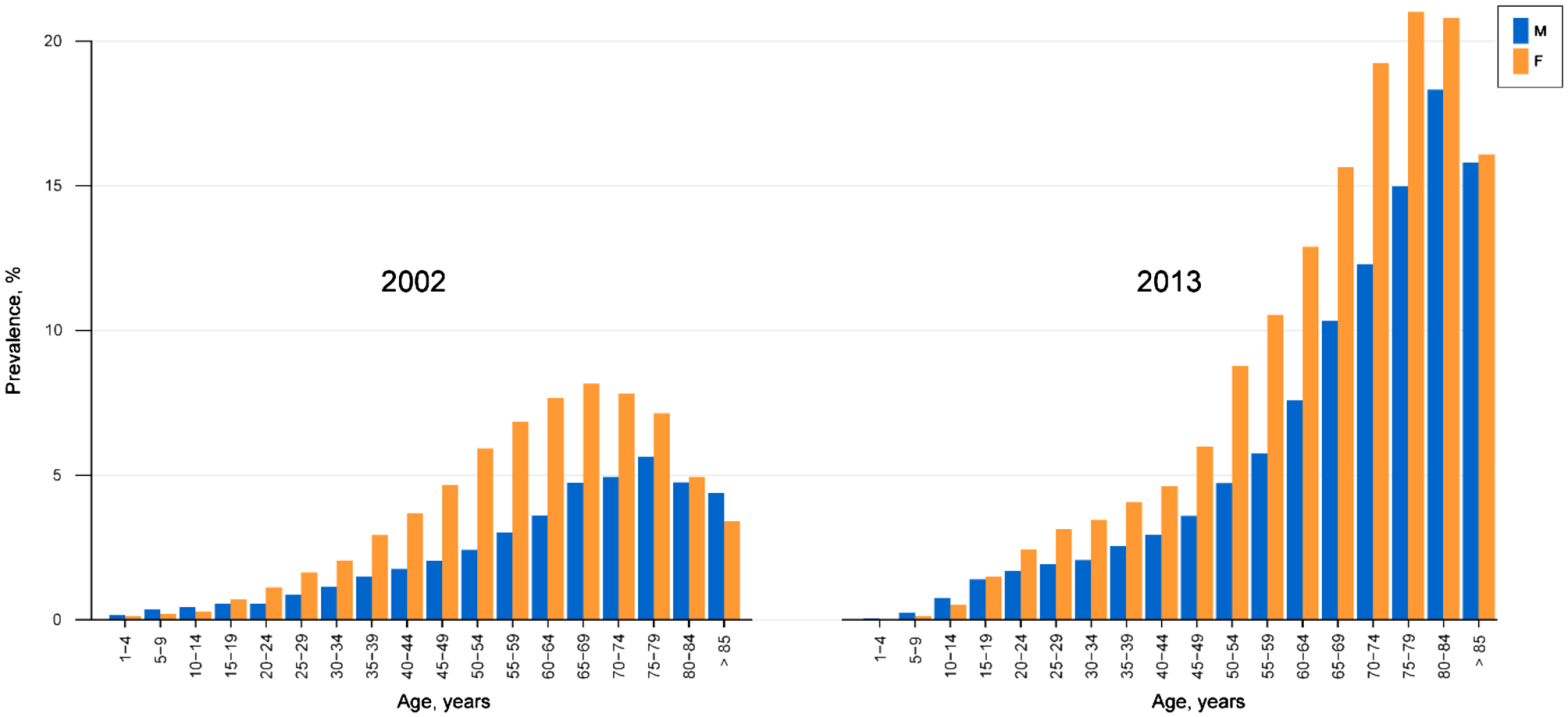
Prevalence of depression based on sex and age in 2002 and 2013.

Figures 2 shows the statistics from 2002 and 2013 as classified by age and sex. The prevalence of depression was higher in females than in males for most age groups. In children under 15 years of age and the elderly over 85 years of age in 2002, males had a higher prevalence than females. In 2013, only male children under 15 years of age had a higher prevalence of depression than female children. As the age of population increased, the prevalence of depression tended to increase. The 65–70 years old group in females and 75–79 years old group in males showed the highest prevalence of depression in 2002. On the other hand, the 75–79 years old group in females and 80–84 years old group in males showed the highest prevalence in 2013.

**Figure 3:**
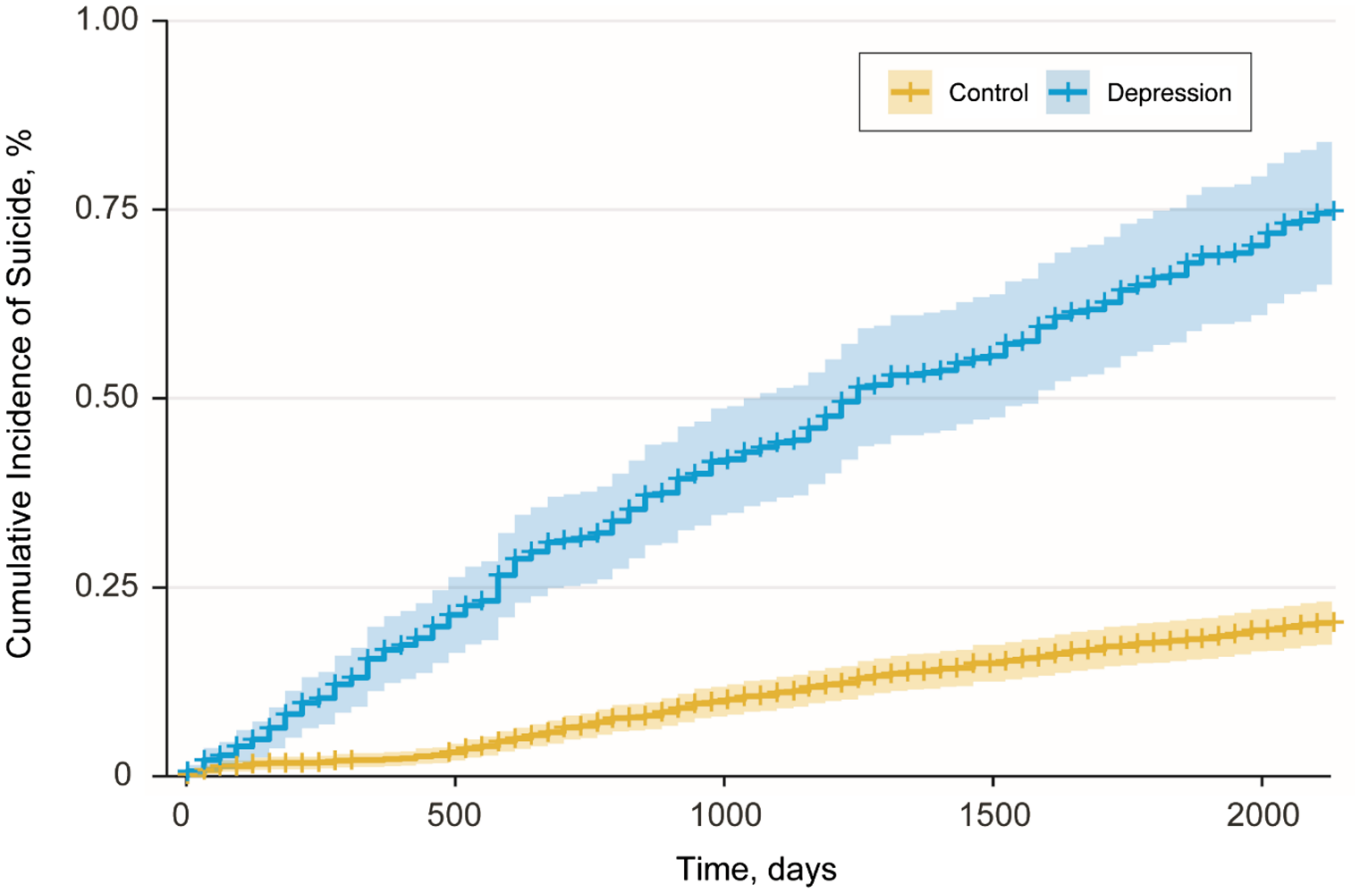
Cumulative incidence of suicide in the population with or without depressive disorder.

**Figure 4:**
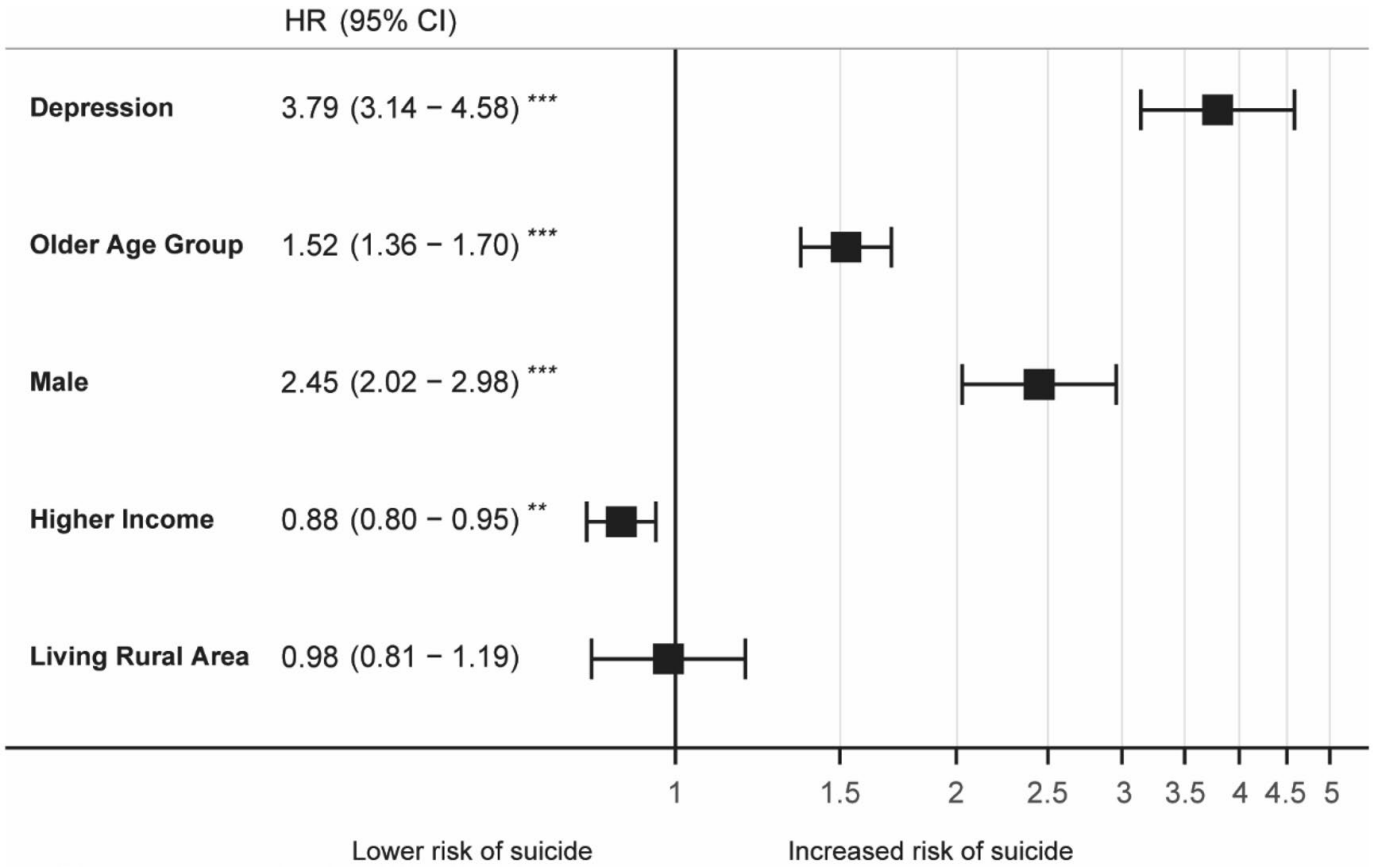
Forest plot of multivariate Cox regression analysis of effect of depression and epidemiological factors on suicide. The number of deaths from suicide according to each factor is listed in Supplementary Table 1.

The cumulative incidence of suicide indicates that the suicide rate of individuals with depressive disorder was significantly higher than that of individuals without the disorder (Fig. 3). Figure 4 and Supplementary Table 1 shows the suicide risk with respect to different population characteristics including depression, age, sex, socioeconomic position, and living area, modelled using Cox's proportional-hazards regression. The suicide risk was significantly higher in people with depression (hazard ratio [HR] 3.79, 95% CI 3.14–4.58) than in those without depression, in older people (HR 1.52, 95% CI 1.36–1.70) than in younger people, and in males (HR 2.45, 95% CI 2.02–2.96) than in females. Higher income groups were at lower suicide risk when compared with lower income groups (HR 0.88, 95% CI 0.80–0.95).

## Discussion

The annual prevalence of depression as estimated by medical records of diagnosis increased during the study period, from 2002 to 2013. It was 3.7% in 2006 and 4.8% in 2011, which is higher than 2.5% in 2006 and 3.1% in 2011 as reported by previous survey studies^7,8^. The annual prevalence of depression was 5.3% in 2013, which was the highest among those reported in South Korea. It is similar to that of high-income (5.5%) and low- to middle-income countries (5.9%) as published in a previous cross-national study^9^. In survey studies, the prevalence could be underrated due to the participants’ nonresponse or underreporting from the fear that disclosing their history of depression may lead to discrimination or avoidance by communities^23^. This use of the claims database in this study makes it free from recall bias or nonresponse. The data was carefully prepared from a database covering the whole South Korean population with systematic stratification to minimize sampling bias^16^. Nevertheless, the data does not include patients who do not visit the clinics or hospitals, allowing for a potential underestimation of depression.

The prevalence of depression has been reported to be lower in Asian countries when compared to Western and Middle Eastern countries^9,10,24–26^. Asian people are known to be reluctant to seek psychiatric treatment due to the stigma of mental illness^27–29^. In Japan, only 5% of the Japanese patients with rheumatoid arthritis had been diagnosed with depression, while 35% of the same population had PHQ-9 scores indicative of depression^30^. Increasing annual prevalence from 2002 to 2013 approaching the level of high-income western countries and decreased population with unmedicated depression (Fig. 1) suggest that although still prevalent, the prejudice against seeking psychiatric treatment has reduced with increasing public awareness of depression^31^.

It is noteworthy that not only did the rate of visits to clinics or hospitals increased, but the rate of antidepressant prescription also increased. Antidepressant prescription rates have also increased in other countries, such as Australia, the USA, and the UK^32–34^. These trends may have been influenced by several factors including public awareness of depression and improvements in antidepressants. Antidepressants such as selective serotonin reuptake inhibitors (SSRIs) and serotonin and norepinephrine reuptake inhibitors (SNRIs) are popular first-line treatments for depression^35^. Newer antidepressants have superior safety profiles and less adverse effects than older tricyclics and monoamine oxidase inhibitors (MAOI)^36^.

The prevalence of depression increased by about 43.82% (3.72% vs 5.35%) in study population from 2005 to 2013. The increase in the prevalence of major depression seems more rapid in South Korea than in the US. Weinberger et al. reported that the prevalence of depression in the US increased by about 8.16% (6.62% vs 7.16%) from 2005 to 2013^37^. The stationary incidence of depression despite increased prevalence of depression suggests that the recovery rate of depressive disorders is low and that depressive disorders might be chronic, lasting years to decades. This finding is more significant if we consider the decrease in the number of unmedicated incidents of depression. South Korea is a highly competitive society that has experienced rapid economic and sociocultural changes over the past few decades. The costs of these rapid changes include occupational stress, rising housing prices, and high divorce rates, all of which are closely associated with depression in South Korea^38–40^. We compared the prevalence of three different categories of depression, i.e., Dx, Dx and Mx, and Dx or Mx, and the overall trends were similar. Interestingly, the prevalence of all three categories have increased rapidly since 2008. The financial crisis that occurred when the Lehman Brothers went bankrupt in 2008 had a worldwide impact. In 2008, mental health problems and suicide rates increased in many countries^41,42^, including South Korea^43,44^.

We observed a higher prevalence of depression in people belonging to the lowest or highest income percentiles than in those of the middle-ranged income percentile. These findings are consistent with previous reports^37,45^ that people of higher economic status are more likely to recognize symptoms of depression, receive adequate treatment for it, and have more positive association towards medication^46,47^.

The prevalence of depressive disorder was higher in women than men in most age groups. It is well known that depression is more prevalent in women than men^6,25,48–50^. Women are susceptible to depression due to sociocultural and biological factors^51^, and they are at a higher risk for major traumas like sexual abuse^52^. Besides, the prior depression and anxiety disorders, social role, poverty, and adverse life events were all regarded as risk factors for depression in women^53^. Our study revealed that boys under 15 years of age had a higher prevalence than girls. Few studies have been conducted on sex differences with respect to the prevalence of depression in children. One study has reported that boys experience more depressive symptoms than girls, and after puberty, the prevalence of depression in women is significantly higher than in men^54^.

In 2013, the prevalence of depression increased with age, being maximum for the 75–79 years old population females and 80–84 years old group in males. The result is consistent with the previous study that reported that aged people have a higher prevalence of depression than younger people in South Korea^49,55^. In a previous study, while the average prevalence of major depressive disorder across all age groups was 3.6%, the prevalence in the subjects who were 65 years or older was 4.6%^55^. Park et al. reported that the prevalence of major depressive disorder at over 65 years of age was 4.93% in a randomly sampled population in a local city area in 2005^56^.

The rapid increase in depression with age is not universal. It has been reported that the risk of major depressive episodes decreased with age in Canada^57^. The authors argued that age seemed to have a protective effect on the incidence of depression^57^. Robert et al. proposed that old age is not an independent risk factor for depression^58^. They suggested that age-related effects on depression were from chronic health problems and related disability. Risk factors such as functional impairment, cognitive problems, and social isolation were associated with higher rates of depression^58^. In South Korea, rapid economic growth and socio-cultural changes have taken place over the last few decades and the elderly have difficulty in adjusting to this abrupt change with a lack of a social support system^59^. The age group showing the highest prevalence of depression, i.e. 60–79 years of age in 2002, increased to over 70 years of age in 2013. This may be due to an increase in the average life expectancy in South Korea^60^. Older people experience more illnesses and more economic difficulties as life expectancy increases. Accordingly, health problems and economic difficulties have been reported to be the main reasons for suicidal thoughts among the older population^61^.

The cumulative incidence of suicide was higher in people with depression than in those without depression. Depression is known to increase suicide risk^3,62^. South Korea has one of the highest suicide rates in the industrialized world^4,5^. The continuously increasing suicide rates in South Korea peaked in 2010^5^. Our study shows that the incidence of depression started to decrease around 2012. South Korea passed the Act for the Prevention of Suicide in 2011 as one of its national suicide prevention policies^5^. The Korea Suicide Prevention Centre was established in 2012, and national suicide prevention projects were promoted by raising public awareness of depression^5^. These national efforts may have contributed to the decreased incidence and prevalence of depression. Indeed, the suicide rates in South Korea have decreased since 2013^63^. Our study shows that the prevalence of depression increased to reach to those of other countries in 2013. However, the treatment of depression by the use of antidepressants also increased (Fig. 1), which might contribute to a decrease in suicide rates since the use of antidepressants contributes to a decrease in suicide rates^64^.

We observed that older people and males had a higher suicide risk than younger people and females (Fig. 4). Older people were more likely to die by suicide than younger people and even if they survived, their prognosis was poor^65^. It is well known that male sex is a risk factor for suicide^66,67^. In our study, people with higher incomes had a lower risk of suicide than those with lower incomes. Suicide risk has been associated with socio-economic factors such as low income and unemployment. To this end, men appear to be more affected by negative economic conditions than women^68^.

There are limitations in this study. Our study could not include people who did not visit clinics or hospital for depressive disorder. The diagnosis of depressive disorder may be omitted to avoid the stigma of mental disorder, or wrongly created by clinicians to reimburse healthcare services. In order to lessen the miscoding error, which is a limitation of claims data, we explored the results with two more auxiliary categories—‘Dx and Mx’ and ‘Dx or Mx’. All three categories showed similar increases in prevalence during the study period.

We analysed a large representative sample of the South Korean population and reported a higher level of prevalence of depression than any previous epidemiological study conducted in South Korea. The prevalence of depression have steadily increased over the last decade with a rapid increase in aged people. Rapid westernization and a competitive social atmosphere in South Korea seem to make people vulnerable to depressive disorders, which is especially true in elderly people who lack a sufficient social support system. We also found that antidepressant prescriptions had increased. The increase in use of healthcare system due to improved economic status or lessening of stigma may be another possible explanation for the observed increase in prevalence of depression. Despite this, the prevalence of depression might still be highly underestimated in South Korea. The relationship between depression and increased suicide risk was confirmed in a large representative sample of the South Korean population.

## Methods

### Data sources and study population

We analysed the NHIS-NSC database of South Korea (2002–2013) which has been implemented by the NHIS of Korea, a single national insurance provider. This cohort consists of a nationally representative random sample of 1,025,340 participants, generated by the NHIS using a systematic, stratified random sampling method from all 46,605,433 individuals from 2002^16^. To maintain the cohort size of approximately one million samples, the NHIS-NSC was renewed annually by adding a representative sample of newborns to compensate for annual disqualifications owing to death and emigration. This database was generated using medical bill expenses of the participants, claimed by medical service providers including hospitals, private clinics, and public centres in South Korea. Depression was diagnosed by physicians, including psychiatrists. The disease diagnosis codes are based on the International Codes of Disease 10th Edition (ICD-10). The database contains subject demographics, disease diagnosis, drug prescription, medical aid beneficiaries, medical bill details, and clinical procedure information generated by all types of medical providers^16^.

Ethical approval was waived in this study by the Institutional Review Board of Asan Medical Center, Seoul, South Korea. Informed consent was waived because the data in the NHIS-NSC were anonymized and de-identified.

### Definition of depression

Depression was defined using the ICD-10 codes for depression (F32.0, F32.1, F32.2, F32.3, F32.8, F32.9, F33.0, F33.1, F33.2, F33.3, F33.4, F33.8, and F33.9). Identification of depression done solely based on diagnosis can be misleading due to the possibility of over-inclusion or misdiagnosis^69^. Therefore, we investigated the prevalence of two more categories of depression. One was the prevalence of depression diagnosis and a history of antidepressant treatment (‘Dx and Mx’ group), and the other was prevalence of depression diagnosis or a history of antidepressant treatment (‘Dx or Mx’ group). The ‘Dx and Mx’ and ‘Dx or Mx’ groups were identified based on the availability of patient claims records with a diagnosis of depression along with records of prescription of antidepressants, and of claims records with a diagnosis of depression or records of prescription of antidepressants, respectively. Here, antidepressants included all in the market available in South Korea during the study period: Selective Serotonin Reuptake Inhibitors, Serotonin-Norepinephrine Reuptake Inhibitors, Norepinephrine-Dopamine Reuptake Inhibitors, Tricyclic antidepressants, Monoamine Oxidase Inhibitors.

### Statistical analyses

We calculated the annual age- and sex-standardized prevalence of depression from 2002 to 2013 and annual age- and sex-standardized incidence of depression from 2005 to 2013, which were standardized based on the cohort structure of 2013 (Supplementary Table 2). Only individuals with no previous diagnosis for depression from 2002 to 2004 were counted as incident cases for the relevant year. The annual prevalence according to sex and age groups was calculated in 2002 and 2013 to visualize the pattern of increased prevalence during the decade. The presence of any linear trends in time for annual prevalence was tested using the chi-square test. Survival analysis was performed using Cox’s proportional models to determine the associations between population characteristics (age group, sex, socioeconomic position measured by income group, living in metropolitan versus non-metropolitan area, and diagnosis of depression) and the risk of committing suicide. We selected cases of incidence in 2008 to analyse the latest five-year suicide for the depressive disorder. Randomly selected non-case controls, subjects with no major depressive disorder, were matched with a ratio of 3:1 to the patients with major depressive disorder from the NHIS-NSC in 2008, according to age and sex (Supplementary Table 3). All statistical analyses were conducted using the R package ver. 3.2.3.

## Supplementary information

Supplementary Tables.

## Data Availability

The data that support the findings of this study are available from the Korea National Health Insurance Service upon reasonable request and with the appropriate review process.

## References

[1] GBD 2017 Disease and Injury Incidence and Prevalence Collaborators (2018). Global, regional, and national incidence, prevalence, and years lived with disability for 354 diseases and injuries for 195 countries and territories, 1990–2017: A systematic analysis for the Global Burden of Disease Study 2017. Lancet.

[2] Bolton JM, Gunnell D, Turecki G (2015). Suicide risk assessment and intervention in people with mental illness. BMJ.

[3] Chesney E, Goodwin GM, Fazel S (2014). Risks of all-cause and suicide mortality in mental disorders: A meta-review. World Psychiatry.

[4] OECD (2015). Health at a Glance 2015: OECD Indicators.

[5] Lee S-U (2018). Changing trends in suicide rates in South Korea from 1993 to 2016: A descriptive study. BMJ Open.

[6] Cho MJ (2007). Lifetime and 12-month prevalence of DSM-IV psychiatric disorders among Korean adults. J. Nerv. Ment. Dis..

[7] Cho MJ (2010). Prevalence of DSM-IV major mental disorders among Korean adults: A 2006 National Epidemiologic Survey (KECA-R). Asian. J. Psychiatr..

[8] Cho MJ (2015). Prevalence and correlates of DSM-IV mental disorders in South Korean adults: The Korean Epidemiologic Catchment Area Study 2011. Psychiatry Investig..

[9] Bromet E (2011). Cross-national epidemiology of DSM-IV major depressive episode. BMC Med..

[10] Lim GY, Tam WW (2018). Prevalence of Depression in the Community from 30 Countries between 1994 and 2014. Sci. Rep..

[11] Hengartner MP, Angst F, Ajdacic-Gross V, Rossler W, Angst J (2016). Treated versus non-treated subjects with depression from a 30-year cohort study: Prevalence and clinical covariates. Eur. Arch. Psychiatry. Clil. Neurosci..

[12] Kohn R, Saxena S, Levav I, Saraceno B (2004). The treatment gap in mental health care. Bull. World Health Organ..

[13] Simon GE, Fleck M, Lucas R, Bushnell DM (2004). Prevalence and predictors of depression treatment in an international primary care study. Am. J. Psychiatry..

[14] Cipriani A, Barbui C, Geddes JR (2005). Suicide, depression, and antidepressants. BMJ.

[15] Dragioti E (2019). Association of antidepressant use with adverse health outcomes: A systematic umbrella review. JAMA Psychiatry.

[16] Lee J, Lee JS, Park SH, Shin SA, Kim K (2017). Cohort profile: The National Health Insurance Service-National Sample Cohort (NHIS-NSC), South Korea. Int. J. Epidemiol..

[17] Lee JH, Lim NK, Cho MC, Park HY (2016). Epidemiology of heart failure in Korea: Present and future. Korean Cir. J..

[18] Rim TH (2018). Incidence and prevalence of uveitis in South Korea: A nationwide cohort study. Br. J. Ophthalmol..

[19] Tae BS, Balpukov U, Cho SY, Jeong CW (2018). Eleven-year cumulative incidence and estimated lifetime prevalence of urolithiasis in Korea: A National Health Insurance Service-National Sample Cohort Based Study. J. Korean Med. Sci..

[20] Krosnick JA (1999). Survey research. Annu. Rev. Psychol..

[21] Simon GE, VonKorff M (1995). Recall of psychiatric history in cross-sectional surveys: Implications for epidemiologic research. Epidemiol Rev..

[22] World Health Organization, Suicide data. https://www.who.int/mental_health/prevention/suicide/suicideprevent/en/. Accesssed 27 Sep 2018.

[23] Kelly CM, Jorm AF (2007). Stigma and mood disorders. Curr. Opin. Psychiatry..

[24] Weissman MM (1996). Cross-national epidemiology of major depression and bipolar disorder. JAMA.

[25] Andrade L (2003). The epidemiology of major depressive episodes: Results from the International Consortium of Psychiatric Epidemiology (ICPE) Surveys. Int. J. Methods Psychiatr. Res..

[26] Chong SA (2012). A population-based survey of mental disorders in Singapore. Ann. Acad. Med. Singapore..

[27] Ng CH (1997). The stigma of mental illness in Asian cultures. Aust. N. Z. J. Psychiatry..

[28] Lauber C, Rössler W (2007). Stigma towards people with mental illness in developing countries in Asia. Int. Rev. Psychiatry..

[29] McDONALD, M. Stressed and Depressed, Koreans Avoid Therapy. The New York Times. https://www.nytimes.com/2011/07/07/world/asia/07iht-psych07.html (2011).

[30] Sruamsiri R, Kaneko Y, Mahlich J (2017). The underrated prevalence of depression in Japanese patients with rheumatoid arthritis—Evidence from a Nationwide survey in Japan. BMC Rheumatol..

[31] Samsung Medical Center (2017). The Survey of Mental Disorders in Korea.

[32] Stephenson CP, Karanges E, McGregor IS (2013). Trends in the utilisation of psychotropic medications in Australia from 2000 to 2011. Aust. N. Z. J. Psychiatry..

[33] Ilyas S, Moncrieff J (2012). Trends in prescriptions and costs of drugs for mental disorders in England, 1998–2010. Br. J. Psychiatry J. Mental Sci..

[34] Olfson M, Marcus SC (2009). National patterns in antidepressant medication treatment. Arch. Gen. Psychiatry.

[35] Woo YS (2017). Korean medication algorithm for depressive disorder 2017(I): Major depressive disorder without psychotic features. Mood Emot..

[36] Westenberg HGM, Sandner C (2006). Tolerability and safety of fluvoxamine and other antidepressants. Int. J. Clin. Pract..

[37] Weinberger AH (2018). Trends in depression prevalence in the USA from 2005 to 2015: Widening disparities in vulnerable groups. Psychol. Med..

[38] Cho JJ (2008). Occupational stress and depression in Korean employees. Int. Arch. Occup. Environ. Health..

[39] Lee T-H (2016). Depressive symptoms of house-poor persons: Korean panel data evidence. Int. J. Soc. Psychiatry..

[40] Kong KA, Choi HY, Kim SI (2017). Mental health among single and partnered parents in South Korea. PLoS ONE.

[41] Chang S-S, Stuckler D, Yip P, Gunnell D (2013). Impact of 2008 global economic crisis on suicide: Time trend study in 54 countries. BMJ.

[42] Barr B, Kinderman P, Whitehead M (2015). Trends in mental health inequalities in England during a period of recession, austerity and welfare reform 2004 to 2013. Soc. Sci. Med..

[43] Kim MY, Jung KH, Kum HS (2011). An explanatory analysis of the economic crisis and suicide rate at the aggregate level: Age- and gender-specific suicide rates at the 15 South Korean provinces, 1992–2009. Korean J. Policy Anal. Eval..

[44] Korean statistical information service, Suicide rate. https://www.index.go.kr/unify/idx-info.do?idxCd=8040. Accessed 12 June 2020.

[45] Chien IC (2007). The prevalence and incidence of treated major depressive disorder among National Health Insurance Enrollees in Taiwan, 1996 to 2003. Can. J. Psychiatry..

[46] von dem Knesebeck O (2013). Socioeconomic status and beliefs about depression, schizophrenia and eating disorders. Soc. Psychiatry Psychiatr. Epidemiol..

[47] Warden D (2009). Income and attrition in the treatment of depression: A STAR*D report. Depress Anxiety..

[48] Kim JI, Choe MA, Chae YR (2009). Prevalence and predictors of geriatric depression in community-dwelling elderly. Asian. Nurs. Res..

[49] Oh DH (2013). Prevalence and correlates of depressive symptoms in korean adults: Results of a 2009 korean community health survey. J. Korean. Med. Sci..

[50] Maier W (1999). Gender differences in the prevalence of depression: A survey in primary care. J. Affect. Disord..

[51] Kuehner C (2003). Gender differences in unipolar depression: An update of epidemiological findings and possible explanations. Acta. Psychiatr. Scand..

[52] Nolen-Hoeksema S (2001). Gender differences in depression. Curr. Dir. Psychol. Sci..

[53] Piccinelli M, Wilkinson G (2000). Gender differences in depression. Crit. Rev. Br. J. Psychiatry..

[54] Twenge JM, Nolen-Hoeksema S (2002). Age, gender, race, socioeconomic status, and birth cohort difference on the children's depression inventory: A meta-analysis. J. Abnorm. Psychol..

[55] Ohayon MM, Hong SC (2006). Prevalence of major depressive disorder in the general population of South Korea. J. Psychiatr. Res..

[56] Park JH (2010). Prevalence of major depressive disorder and minor depressive disorder in an elderly Korean population: Results from the Korean Longitudinal Study on Health and Aging (KLoSHA). J. Affect. Disord..

[57] Wade TJ, Cairney J (2000). The effect of sociodemographics, social stressors, health status and psychosocial resources on the age-depression relationship. Can. J. Public Health..

[58] Roberts RE, Kaplan GA, Shema SJ, Strawbridge WJ (1997). Does growing old increase the risk for depression?. Am. J. Psychiatry..

[59] Choe HS, Ryu YK (2003). A study on the levels, trends, and composition of the old-age poverty in Korea. J. Korea Gerontol. Society..

[60] Korean statistical information service. Life expectancy. https://kosis.kr/statHtml/statHtml.do?orgId=101&tblId=DT_1B42&vw_cd=MT_ZTITLE&list_id=F_29&seqNo=&lang_mode=ko&language=kor&obj_var_id=&itm_id=&conn_path=MT_ZTITLE. Accessed 12 June 2020.

[61] Korean statistical information service. Reasons and attempt to think suicide by general feature of older persons. https://kosis.kr/statHtml/statHtml.do?orgId=117&tblId=DT_117071_013&vw_cd=MT_ETITLE&list_id=117_11771_003_04&scrId=&seqNo=&language=en&obj_var_id=&itm_id=&conn_path=A6&path=%252Feng%252Fsearch%252FsearchList.do. Accessed 12 June 2020.

[62] Choi JW, Lee KS, Kim TH, Choi J, Han E (2019). Suicide risk after discharge from psychiatric care in South Korea. J. Affect. Disord..

[63] Korea Suicide Prevention Center. Suicide status. https://spckorea-stat.or.kr/korea01.do. Accessed 12 June 2020.

[64] Rihmer Z (2001). Can better recognition and treatment of depression reduce suicide rates? A brief review. Eur. Psychiatry..

[65] Rodda J, Walker Z, Carter J (2011). Depression in older adults. BMJ.

[66] Crump C, Sundquist K, Sundquist J, Winkleby MA (2013). Sociodemographic, psychiatric and somatic risk factors for suicide: A Swedish national cohort study. Psychol. Med..

[67] Hawton K, Comabella C, Haw C, Saunders K (2013). Risk factors for suicide in individuals with depression: A systematic review. J. Affect. Disord..

[68] Qin P, Agerbo E, Mortensen PB (2003). Suicide risk in relation to socioeconomic, demographic, psychiatric, and familial factors: A national register-based study of all suicides in Denmark, 1981–1997. Am. J. Psychiatry..

[69] Mitchell AJ, Vaze A, Rao S (2009). Clinical diagnosis of depression in primary care: A meta-analysis. Lancet.

