# Supplementary material for: Increased prevalence of depression in South Korea from 2002 to 2013
Source: Sci Rep. 2020 Oct 12;10:16979. doi: 10.1038/s41598-020-74119-4 (PMC7550589; doi:10.1038/s41598-020-74119-4)
Supplement: Supplementary file 1 — Supplementary Tables. [file 41598_2020_74119_MOESM1_ESM.docx]

**Increased prevalence of depression in South Korea from 2002 to 2013**

Ga Eun Kim^1^, Min-Woo Jo^2*^, Yong-Wook Shin^3*^

^1^Department of Psychiatry, College of Medicine, Ewha Womans University, Seoul, Korea.

^2^Department of Preventive Medicine, University of Ulsan College of Medicine, Seoul, Korea.

^3^Department of Psychiatry, Asan Medical Center, University of Ulsan College of Medicine, Seoul, Korea.

Supplementary Table 1. Number of suicides according to population characteristics

|  | Participants | Suicide (%) |
| --- | --- | --- |
| Diagnosis |  |  |
| Control | 99408 | 196 (0.20%) |
| Depression | 33136 | 239 (0.72%) |
| Sex |  |  |
| Male | 46064 | 236 (0.51%) |
| Female | 86480 | 199 (0.23%) |
| Age |  |  |
| 0–9 | 4780 | 4 (0.08%) |
| 20–39 | 23188 | 61 (0.26%) |
| 40–59 | 50336 | 139 (0.28%) |
| 60–79 | 47488 | 190 (0.40%) |
| > 80 | 6752 | 41 (0.61%) |
| Household income, percentile, n |  |  |
| < 20% | 25907 | 96 (0.37%) |
| 21–50% | 29388 | 108 (0.37%) |
| 51–80% | 38626 | 117 (0.30%) |
| > 81% | 38623 | 114 (0.30%) |
| Residential area, n |  |  |
| Metropolitan | 62570 | 202 (0.32%) |
| Nonmetropolitan | 69974 | 233 (0.33%) |

* Income group was divided into 4 groups according to the information of insurance premium group: Group 1 included people from Medicaid and 20%tile of insurance premium; Group 2 included people of 21~50%tile of insurance premium; Group 3 included people of 51~80%tile of insurance premium; Group 4 included people of 81~100%tile of insurance premium;

** The region was divided into two groups, metropolitan and countryside. Metropolitan included the capital city of Korea (Seoul) and six metropolitan cities such as Busan, Incheon, Daegu, Daejeon, Gwangju, and Ulsan. Other regions were allocated in the nonmetropolitan.

Supplementary Table 2. Number of total population and the patients with depression in each year

| Year | Number of subjects in total population | Number of patients newly diagnosed with depression | Number of patients diagnosed with depression | Number of patients in ‘Dx and Mx’ group | Number of patients in ‘Dx or Mx’ group |
| --- | --- | --- | --- | --- | --- |
| 2002 | 1025340 |  | 23235 | 6417 | 33460 |
| 2003 | 1017468 |  | 26671 | 8624 | 39307 |
| 2004 | 1016580 |  | 30002 | 9771 | 42891 |
| 2005 | 1016820 | 16741 | 32699 | 10561 | 46305 |
| 2006 | 1002005 | 14676 | 32146 | 10263 | 45760 |
| 2007 | 1020743 | 12815 | 30607 | 11355 | 45319 |
| 2008 | 1000785 | 14199 | 33136 | 14520 | 47303 |
| 2009 | 998527 | 15305 | 37460 | 17315 | 50311 |
| 2010 | 1002031 | 14920 | 38993 | 18849 | 50856 |
| 2011 | 1006481 | 17518 | 45816 | 23329 | 58225 |
| 2012 | 1011123 | 19028 | 52743 | 28796 | 63956 |
| 2013 | 1014730 | 17349 | 54250 | 29163 | 65075 |

Supplementary Table 3. Demographic factors of depression and no depression group of 2008 for survival analysis

|  | Depression group | Control group | p | Effect Size  (Cramer’s V) |
| --- | --- | --- | --- | --- |
| Sex |  |  | 1 | 0 |
| Male | 11516 (34.8%) | 34548 (34.8%) |  |  |
| Female | 21620 (65.2%) | 64860 (65.2%) |  |  |
| Age |  |  | 1 | 0 |
| 0–19 | 1195 (3.6%) | 3585 (3.6%) |  |  |
| 20–39 | 5797 (17.5%) | 17391 (17.5%) |  |  |
| 40–59 | 12584 (38.0%) | 37752 (38.0%) |  |  |
| 60–79 | 11872 (35.8%) | 35616 (35.8%) |  |  |
| > 80 | 1688 (5.1%) | 5064 (5.1%) |  |  |
| Household income, percentile, n (%) |  |  | <0.001 | 0.03 |
| < 20% | 5940 (17.9%) | 19967 (20.1%) |  |  |
| 21–50% | 7153 (21.6%) | 22235 (22.4%) |  |  |
| 51–80% | 9638 (29.1%) | 28988 (29.2%) |  |  |
| > 81% | 10405 (31.4%) | 28218 (28.4%) |  |  |
| Residential area, n (%) |  |  | 0.04 | 0.007 |
| Metropolitan | 15443 (46.6%) | 47127 (47.4%) |  |  |
| Nonmetropolitan | 17693 (53.4%) | 52281 (52.6%) |  |  |

* Income group was divided into 4 groups according to the information of insurance premium group: Group 1 included people from Medicaid and 20%tile of insurance premium; Group 2 included people of 21–50%tile of insurance premium; Group 3 included people of 51–80%tile of insurance premium; Group 4 included people of 81–100%tile of insurance premium;

** The region was divided into two groups, metropolitan and countryside. Metropolitan included the capital city of Korea (Seoul) and six metropolitan cities such as Busan, Incheon, Daegu, Daejeon, Gwangju, and Ulsan. Other regions were allocated in the nonmetropolitan.
